# Poly[[[[1-ethyl-6,8-difluoro-7-(3-methyl­piperazin-1-yl)-4-oxo-1,4-dihydro­quinoline-3-carboxyl­ato]cadmium]-μ-benzene-1,4-dicarboxyl­ato] trihydrate]

**DOI:** 10.1107/S160053681004328X

**Published:** 2010-10-31

**Authors:** Xin-Ping Kang, Zhe An, Rena Kasimu

**Affiliations:** a School of Pharmacological Sciences, Xinjiang Medical University, Urumqi 830054, People’s Republic of China; b School of Chemistry and Life Science, Guangdong University of Petrochemical Technology, Maoming 525000, People’s Republic of China

## Abstract

In the title layered coordination polymer, {[Cd(C_17_H_18_F_2_N_3_O_3_)(C_8_H_4_O_4_)]·3H_2_O}_*n*_, the Cd^II^ atom exhibits a very distorted CdO_6_ octa­hedral geometry defined by one *O* ^3^,*O* ^4^-bidentate 1-ethyl-6,8-difluoro-7-(3-methyl­piperazin-1-yl)-4-oxo-1,4-dihydro­quinoline-3-carboxyl­ate (lome) ligand, one *O*,*O*′-bidentate benzene-1,4-dicarboxyl­ate (bdc) dianion and two *O*-monodentate bdc dianions. Both the bdc species in the asymmetric unit are completed by crystallographic inversion symmetry. The bridging bdc dianions link the cadmium nodes into a recta­ngular grid lying parallel to (01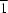). A network of N—H⋯O and O—H⋯O hydrogen bonds helps to establish the packing.

## Related literature

For background on the medicinal uses of lomefloxacin, see: Mizuki *et al.* (1996 ▶).
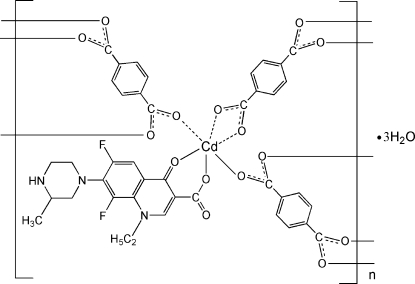


## Experimental

### 

#### Crystal data

[Cd(C_17_H_18_F_2_N_3_O_3_)(C_8_H_4_O_4_)]·3H_2_O
*M* *_r_* = 680.90
Triclinic, 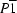

*a* = 9.7924 (7) Å
*b* = 11.9788 (8) Å
*c* = 13.3981 (9) Å
α = 114.138 (1)°
β = 103.430 (1)°
γ = 100.295 (1)°
*V* = 1327.00 (16) Å^3^
*Z* = 2
Mo *K*α radiation
μ = 0.90 mm^−1^
*T* = 295 K
0.32 × 0.24 × 0.18 mm

#### Data collection

Bruker SMART CCD diffractometer
Absorption correction: multi-scan (*SADABS*; Bruker, 2001 ▶) *T*_min_ = 0.762, *T*_max_ = 0.855
6581 measured reflections
4624 independent reflections
4108 reflections with *I* > 2σ(*I*)
*R*_int_ = 0.014

#### Refinement

*R*[*F* ^2^ > 2σ(*F* ^2^)] = 0.044
*wR*(*F* ^2^) = 0.135
*S* = 1.11
4624 reflections
372 parameters
H-atom parameters constrained
Δρ_max_ = 1.76 e Å^−3^
Δρ_min_ = −0.81 e Å^−3^

### 

Data collection: *APEX2* (Bruker, 2004 ▶); cell refinement: *SAINT-Plus* (Bruker, 2001 ▶); data reduction: *SAINT-Plus*; program(s) used to solve structure: *SHELXS97* (Sheldrick, 2008 ▶); program(s) used to refine structure: *SHELXL97* (Sheldrick, 2008 ▶); molecular graphics: *SHELXTL* (Sheldrick, 2008 ▶); software used to prepare material for publication: *SHELXTL*.

## Supplementary Material

Crystal structure: contains datablocks I, global. DOI: 10.1107/S160053681004328X/hb5699sup1.cif

Structure factors: contains datablocks I. DOI: 10.1107/S160053681004328X/hb5699Isup2.hkl

Additional supplementary materials:  crystallographic information; 3D view; checkCIF report

## Figures and Tables

**Table 1:** Selected bond lengths (Å)

Cd1—O6	2.213 (4)
Cd1—O3	2.238 (3)
Cd1—O7^i^	2.295 (4)
Cd1—O1	2.299 (4)
Cd1—O5	2.322 (4)
Cd1—O4	2.510 (4)

Symmetry code: (i) 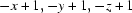.

**Table 2:** Hydrogen-bond geometry (Å, °)

*D*—H⋯*A*	*D*—H	H⋯*A*	*D*⋯*A*	*D*—H⋯*A*
N3—H3*A*⋯O8	0.86	2.33	2.749 (8)	110
O8—H8*A*⋯O2^ii^	0.85	2.02	2.854 (7)	166
O8—H8*B*⋯O4^iii^	0.85	2.01	2.837 (7)	166
O9—H9*B*⋯O10^iv^	0.85	1.84	2.687 (14)	178
O9—H9*A*⋯O5^v^	0.85	1.93	2.784 (9)	178
O10—H5O⋯O2	0.85	2.13	2.971 (10)	171
O10—H6O⋯O9	0.85	2.42	2.957 (14)	121

Symmetry codes: (ii) 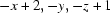; (iii) 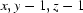; (iv) 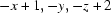; (v) 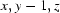.

**Fig. 1.**
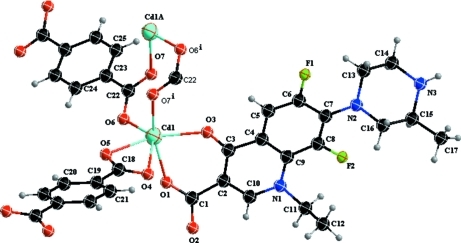
The asymmetric unit of (I), expanded to show the Cd coordination, showing 50% displacement ellipsoids. Symmetry code: (i) x, y, z.

**Fig. 2.**
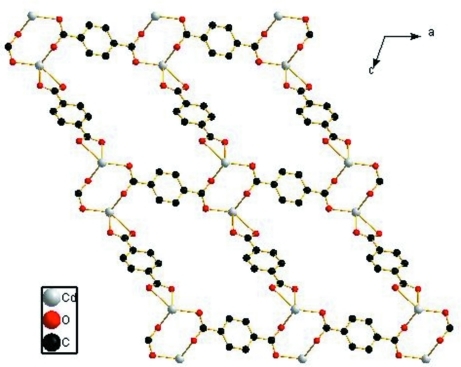
A view of part of a two-dimensional polymeric sheet in (I) showing the square-grid connectivity (lomefloxacin molecule, H atoms and water molecule have been omitted for clarity).

## supplementary crystallographic information

### Comment

Lomefloxacin (*H*-Lome,1,4-dihydro-6,8-difluoro-1-ethyl-7- (3-methyl-1-piperazinyl)-4-oxo-3-quinoline carboxylic acid) is a new member of the class of quinolones that is used to treat infections (Mizuki *et al.*, 1996). The metal complexes of lomefloxacin have not been reported.

The title cadmium(II)-containing complex of lomefloxacin, (I), is reported here (Fig. 1).

The structure of (I) is built up from Cd^2+^ cations, lome ligands, 1,4-benzenedicarboxylate anions, three uncoordinated water molecules (Fig. 1). The Cd atom exhibits a distorted CdO_6_ octahedral geometry, two O atom come from one bidentate O,*O*-bonded 1,4-dihydro-6,8-difluoro-1-ethyl-7-(3-methyl-1-piperazinyl)-4-oxo-3-quinoline carboxylic (lome) and four O atom come from three 1,4-benzenedicarboxylic acid molecules.

In title compound (I) form a square grid propagating in (Fig. 2) which 1,4-benzenedicarboxylic acid is bridged ligands.

The components of (I) are linked by O—H···O and O—H···N hydrogen bonds involving all the potential donors, generating a three-dimensional supramolecular network.

### Experimental

A mixture of Cd(NO_3_)_2_.2H_2_O (0.5 mmol), lomefloxacin (0.6 mmol), 1,4-benzenedicarboxylic acid (0.25 mmol), and water (12 ml) was stirred for 30 min in air. The mixture was then transferred to a 25 ml Teflon reactor and kept at 433 K for 72 h under autogenous pressure. Colourless single crystals of (I) suitable for X-ray analysis were obtained from the reaction mixture.

### Refinement

The H on the C atoms and N atoms were positioned geometrically (C—H = 0.93–0.97 Å, N—H = 0.86 Å) and refined as riding with *U*_iso_(H) = 1.2*U*_eq_(C or N). The H atoms on water molecules were placed at chemically sensible positions on the basis of hydrogen bonds, but these were not refined, O—H = 0.85 Å and U(H) = 1.5Ueq(O).

### Crystal data

**Table:**

[Cd(C_17_H_18_F_2_N_3_O_3_)(C_8_H_4_O_4_)]·3H_2_O	*Z* = 2
*M**_r_* = 680.90	*F*(000) = 690
Triclinic, *P*1	*D*_x_ = 1.704 Mg m^−^^3^
Hall symbol: -P 1	Mo *K*α radiation, λ = 0.71073 Å
*a* = 9.7924 (7) Å	Cell parameters from 6498 reflections
*b* = 11.9788 (8) Å	θ = 1.8–25.0°
*c* = 13.3981 (9) Å	µ = 0.90 mm^−^^1^
α = 114.138 (1)°	*T* = 295 K
β = 103.430 (1)°	Prism, colourless
γ = 100.295 (1)°	0.32 × 0.24 × 0.18 mm
*V* = 1327.00 (16) Å^3^	

### Data collection

**Table:**

Bruker SMART CCD diffractometer	4624 independent reflections
Radiation source: fine-focus sealed tube	4108 reflections with *I* > 2σ(*I*)
graphite	*R*_int_ = 0.014
ω scans	θ_max_ = 25.0°, θ_min_ = 1.8°
Absorption correction: multi-scan (*SADABS*; Bruker, 2001)	*h* = −11→11
*T*_min_ = 0.762, *T*_max_ = 0.855	*k* = −14→13
6581 measured reflections	*l* = −15→15

### Refinement

**Table:**

Refinement on *F*^2^	Primary atom site location: structure-invariant direct methods
Least-squares matrix: full	Secondary atom site location: difference Fourier map
*R*[*F*^2^ > 2σ(*F*^2^)] = 0.044	Hydrogen site location: inferred from neighbouring sites
*wR*(*F*^2^) = 0.135	H-atom parameters constrained
*S* = 1.11	*w* = 1/[σ^2^(*F*_o_^2^) + (0.077*P*)^2^ + 3.3034*P*] where *P* = (*F*_o_^2^ + 2*F*_c_^2^)/3
4624 reflections	(Δ/σ)_max_ = 0.001
372 parameters	Δρ_max_ = 1.76 e Å^−^^3^
0 restraints	Δρ_min_ = −0.81 e Å^−^^3^

### Special details

**Table:**

Geometry. All e.s.d.'s (except the e.s.d. in the dihedral angle between two l.s. planes) are estimated using the full covariance matrix. The cell e.s.d.'s are taken into account individually in the estimation of e.s.d.'s in distances, angles and torsion angles; correlations between e.s.d.'s in cell parameters are only used when they are defined by crystal symmetry. An approximate (isotropic) treatment of cell e.s.d.'s is used for estimating e.s.d.'s involving l.s. planes.
Refinement. Refinement of *F*^2^ against ALL reflections. The weighted *R*-factor *wR* and goodness of fit *S* are based on *F*^2^, conventional *R*-factors *R* are based on *F*, with *F* set to zero for negative *F*^2^. The threshold expression of *F*^2^ > σ(*F*^2^) is used only for calculating *R*-factors(gt) *etc*. and is not relevant to the choice of reflections for refinement. *R*-factors based on *F*^2^ are statistically about twice as large as those based on *F*, and *R*- factors based on ALL data will be even larger.

### Fractional atomic coordinates and isotropic or equivalent isotropic displacement parameters (Å^2^)

**Table:**

	*x*	*y*	*z*	*U*_iso_*/*U*_eq_	
Cd1	0.60296 (4)	0.51272 (3)	0.66873 (3)	0.02772 (15)	
F1	0.7760 (5)	0.0511 (4)	0.2452 (3)	0.0572 (10)	
F2	0.7085 (5)	−0.1943 (3)	0.4378 (3)	0.0528 (9)	
N1	0.6840 (5)	0.0195 (4)	0.6257 (4)	0.0319 (9)	
N2	0.7709 (6)	−0.1778 (5)	0.2569 (4)	0.0469 (12)	
N3	0.7833 (7)	−0.4099 (5)	0.0884 (5)	0.0608 (16)	
H3A	0.8174	−0.4574	0.0387	0.073*	
O1	0.5949 (5)	0.4247 (4)	0.7917 (3)	0.0419 (9)	
O2	0.6845 (5)	0.3373 (4)	0.8987 (3)	0.0499 (11)	
O3	0.6497 (5)	0.3294 (3)	0.5729 (3)	0.0384 (9)	
O4	0.8541 (4)	0.6499 (4)	0.8163 (4)	0.0457 (10)	
O5	0.6668 (4)	0.7265 (4)	0.8070 (4)	0.0501 (11)	
O6	0.3731 (4)	0.5173 (4)	0.6448 (4)	0.0480 (10)	
O7	0.3361 (4)	0.4186 (4)	0.4565 (3)	0.0381 (9)	
O8	1.0201 (6)	−0.3309 (6)	0.0299 (5)	0.0805 (17)	
H8A	1.1082	−0.3335	0.0399	0.121*	
H8B	0.9839	−0.3370	−0.0370	0.121*	
O9	0.4490 (9)	−0.1577 (9)	0.8478 (8)	0.147 (4)	
H9A	0.5155	−0.1934	0.8367	0.220*	
H9B	0.4402	−0.1481	0.9123	0.220*	
O10	0.5836 (11)	0.1231 (9)	0.9489 (9)	0.142 (3)	
H5O	0.6181	0.1897	0.9428	0.213*	
H6O	0.5537	0.0588	0.8811	0.213*	
C1	0.6442 (6)	0.3397 (5)	0.8035 (4)	0.0346 (12)	
C2	0.6576 (6)	0.2330 (5)	0.6991 (4)	0.0303 (11)	
C3	0.6622 (6)	0.2386 (5)	0.5956 (4)	0.0302 (11)	
C4	0.6902 (6)	0.1292 (5)	0.5103 (4)	0.0294 (10)	
C5	0.7112 (6)	0.1352 (5)	0.4121 (5)	0.0360 (12)	
H5	0.7033	0.2059	0.4011	0.043*	
C6	0.7428 (7)	0.0384 (6)	0.3335 (5)	0.0396 (13)	
C7	0.7459 (6)	−0.0757 (5)	0.3398 (4)	0.0363 (12)	
C8	0.7193 (6)	−0.0807 (5)	0.4357 (4)	0.0344 (12)	
C9	0.6984 (6)	0.0206 (5)	0.5248 (4)	0.0298 (10)	
C10	0.6692 (6)	0.1241 (5)	0.7075 (4)	0.0324 (11)	
H10	0.6666	0.1227	0.7760	0.039*	
C11	0.7087 (7)	−0.0836 (5)	0.6569 (5)	0.0432 (14)	
H11A	0.6522	−0.1672	0.5909	0.052*	
H11B	0.6741	−0.0756	0.7210	0.052*	
C12	0.8695 (7)	−0.0743 (6)	0.6911 (6)	0.0514 (16)	
H12A	0.9038	−0.0823	0.6276	0.077*	
H12B	0.8822	−0.1420	0.7096	0.077*	
H12C	0.9252	0.0075	0.7578	0.077*	
C13	0.7198 (10)	−0.2110 (8)	0.1326 (5)	0.075 (3)	
H13A	0.6421	−0.1739	0.1176	0.090*	
H13B	0.8009	−0.1747	0.1139	0.090*	
C14	0.6651 (10)	−0.3490 (8)	0.0593 (6)	0.077 (2)	
H14A	0.6393	−0.3686	−0.0218	0.092*	
H14B	0.5769	−0.3844	0.0712	0.092*	
C15	0.8317 (11)	−0.3763 (7)	0.2137 (7)	0.072 (2)	
H15	0.7487	−0.4155	0.2302	0.086*	
C16	0.8786 (9)	−0.2384 (7)	0.2861 (6)	0.065 (2)	
H16A	0.9699	−0.2010	0.2789	0.078*	
H16B	0.8990	−0.2196	0.3666	0.078*	
C17	0.9593 (11)	−0.4326 (8)	0.2388 (8)	0.076 (3)	
H17A	1.0309	−0.4109	0.2061	0.113*	
H17B	0.9204	−0.5244	0.2044	0.113*	
H17C	1.0053	−0.3972	0.3213	0.113*	
C18	0.8017 (6)	0.7408 (5)	0.8452 (4)	0.0354 (12)	
C19	0.9057 (6)	0.8760 (5)	0.9286 (4)	0.0316 (11)	
C20	0.8504 (6)	0.9742 (5)	0.9815 (5)	0.0378 (12)	
H20	0.7497	0.9570	0.9688	0.045*	
C21	0.9451 (6)	1.0990 (5)	1.0537 (5)	0.0368 (12)	
H21	0.9081	1.1652	1.0898	0.044*	
C22	0.2939 (5)	0.4741 (5)	0.5409 (5)	0.0325 (11)	
C23	0.1424 (5)	0.4880 (5)	0.5192 (4)	0.0282 (10)	
C24	0.1114 (6)	0.5848 (5)	0.6051 (5)	0.0343 (11)	
H24	0.1860	0.6422	0.6753	0.041*	
C25	−0.0300 (5)	0.5958 (5)	0.5861 (5)	0.0332 (11)	
H25	−0.0498	0.6596	0.6442	0.040*	

### Atomic displacement parameters (Å^2^)

**Table:**

	*U*^11^	*U*^22^	*U*^33^	*U*^12^	*U*^13^	*U*^23^
Cd1	0.0276 (2)	0.0200 (2)	0.0276 (2)	0.00944 (14)	0.00617 (15)	0.00477 (15)
F1	0.098 (3)	0.053 (2)	0.045 (2)	0.041 (2)	0.045 (2)	0.0264 (18)
F2	0.089 (3)	0.0242 (16)	0.0424 (19)	0.0259 (17)	0.0203 (18)	0.0111 (15)
N1	0.041 (2)	0.025 (2)	0.029 (2)	0.0112 (18)	0.0114 (19)	0.0126 (18)
N2	0.069 (3)	0.038 (3)	0.029 (2)	0.032 (3)	0.015 (2)	0.006 (2)
N3	0.093 (5)	0.036 (3)	0.052 (3)	0.032 (3)	0.040 (3)	0.005 (3)
O1	0.062 (3)	0.035 (2)	0.035 (2)	0.0243 (19)	0.0226 (19)	0.0142 (17)
O2	0.077 (3)	0.047 (2)	0.025 (2)	0.030 (2)	0.017 (2)	0.0125 (18)
O3	0.063 (3)	0.0288 (19)	0.035 (2)	0.0267 (18)	0.0252 (19)	0.0163 (17)
O4	0.047 (2)	0.0251 (19)	0.044 (2)	0.0065 (17)	0.0060 (19)	0.0040 (17)
O5	0.035 (2)	0.030 (2)	0.052 (3)	0.0015 (17)	−0.0012 (19)	0.0012 (19)
O6	0.031 (2)	0.063 (3)	0.044 (2)	0.0183 (19)	0.0057 (18)	0.022 (2)
O7	0.036 (2)	0.038 (2)	0.052 (2)	0.0202 (17)	0.0229 (18)	0.0239 (19)
O8	0.065 (3)	0.116 (5)	0.087 (4)	0.031 (3)	0.023 (3)	0.072 (4)
O9	0.108 (6)	0.150 (8)	0.122 (7)	0.065 (6)	0.011 (5)	0.014 (6)
O10	0.150 (8)	0.133 (8)	0.190 (10)	0.070 (6)	0.079 (7)	0.095 (8)
C1	0.041 (3)	0.027 (3)	0.030 (3)	0.011 (2)	0.013 (2)	0.007 (2)
C2	0.035 (3)	0.027 (3)	0.024 (2)	0.011 (2)	0.009 (2)	0.006 (2)
C3	0.035 (3)	0.023 (2)	0.028 (3)	0.012 (2)	0.010 (2)	0.007 (2)
C4	0.036 (3)	0.022 (2)	0.026 (2)	0.012 (2)	0.009 (2)	0.006 (2)
C5	0.052 (3)	0.029 (3)	0.036 (3)	0.022 (2)	0.021 (3)	0.017 (2)
C6	0.055 (3)	0.041 (3)	0.028 (3)	0.023 (3)	0.020 (2)	0.014 (2)
C7	0.046 (3)	0.028 (3)	0.024 (3)	0.018 (2)	0.006 (2)	0.002 (2)
C8	0.046 (3)	0.020 (2)	0.030 (3)	0.015 (2)	0.008 (2)	0.007 (2)
C9	0.036 (3)	0.023 (2)	0.026 (2)	0.011 (2)	0.007 (2)	0.009 (2)
C10	0.044 (3)	0.025 (3)	0.023 (2)	0.011 (2)	0.010 (2)	0.008 (2)
C11	0.072 (4)	0.026 (3)	0.039 (3)	0.019 (3)	0.020 (3)	0.019 (2)
C12	0.064 (4)	0.047 (4)	0.050 (4)	0.029 (3)	0.014 (3)	0.028 (3)
C13	0.100 (6)	0.076 (5)	0.027 (3)	0.057 (5)	0.005 (3)	0.002 (3)
C14	0.101 (6)	0.071 (5)	0.045 (4)	0.048 (5)	0.023 (4)	0.008 (4)
C15	0.109 (7)	0.055 (4)	0.081 (5)	0.047 (5)	0.057 (5)	0.037 (4)
C16	0.084 (5)	0.058 (4)	0.040 (3)	0.050 (4)	0.008 (3)	0.005 (3)
C17	0.127 (7)	0.062 (5)	0.089 (6)	0.069 (5)	0.069 (6)	0.049 (5)
C18	0.043 (3)	0.028 (3)	0.025 (3)	0.004 (2)	0.008 (2)	0.008 (2)
C19	0.036 (3)	0.026 (3)	0.022 (2)	0.003 (2)	0.003 (2)	0.007 (2)
C20	0.032 (3)	0.032 (3)	0.035 (3)	0.003 (2)	0.005 (2)	0.009 (2)
C21	0.041 (3)	0.026 (3)	0.032 (3)	0.010 (2)	0.009 (2)	0.006 (2)
C22	0.027 (3)	0.029 (3)	0.043 (3)	0.007 (2)	0.008 (2)	0.021 (2)
C23	0.025 (2)	0.029 (2)	0.034 (3)	0.010 (2)	0.011 (2)	0.016 (2)
C24	0.027 (3)	0.034 (3)	0.031 (3)	0.005 (2)	0.006 (2)	0.009 (2)
C25	0.029 (3)	0.032 (3)	0.033 (3)	0.013 (2)	0.011 (2)	0.010 (2)

### Geometric parameters (Å, °)

**Table:**

Cd1—O6	2.213 (4)	C5—H5	0.9300
Cd1—O3	2.238 (3)	C6—C7	1.408 (8)
Cd1—O7^i^	2.295 (4)	C7—C8	1.391 (8)
Cd1—O1	2.299 (4)	C8—C9	1.398 (7)
Cd1—O5	2.322 (4)	C10—H10	0.9300
Cd1—O4	2.510 (4)	C11—C12	1.504 (9)
F1—C6	1.356 (6)	C11—H11A	0.9700
F2—C8	1.358 (6)	C11—H11B	0.9700
N1—C10	1.345 (6)	C12—H12A	0.9600
N1—C9	1.397 (7)	C12—H12B	0.9600
N1—C11	1.496 (6)	C12—H12C	0.9600
N2—C7	1.383 (7)	C13—C14	1.447 (11)
N2—C16	1.451 (8)	C13—H13A	0.9700
N2—C13	1.477 (8)	C13—H13B	0.9700
N3—C15	1.489 (10)	C14—H14A	0.9700
N3—C14	1.534 (10)	C14—H14B	0.9700
N3—H3A	0.8600	C15—C16	1.447 (10)
O1—C1	1.251 (6)	C15—C17	1.555 (11)
O2—C1	1.258 (7)	C15—H15	0.9800
O3—C3	1.261 (6)	C16—H16A	0.9700
O4—C18	1.243 (7)	C16—H16B	0.9700
O5—C18	1.253 (7)	C17—H17A	0.9600
O6—C22	1.255 (7)	C17—H17B	0.9600
O7—C22	1.261 (6)	C17—H17C	0.9600
O7—Cd1^i^	2.295 (4)	C18—C19	1.520 (7)
O8—H8A	0.8500	C19—C20	1.380 (8)
O8—H8B	0.8501	C19—C21^ii^	1.380 (8)
O9—H9A	0.8501	C20—C21	1.391 (8)
O9—H9B	0.8500	C20—H20	0.9300
O10—H5O	0.8523	C21—C19^ii^	1.380 (8)
O10—H6O	0.8530	C21—H21	0.9300
C1—C2	1.513 (7)	C22—C23	1.497 (7)
C2—C10	1.377 (7)	C23—C25^iii^	1.391 (7)
C2—C3	1.427 (7)	C23—C24	1.399 (7)
C3—C4	1.466 (7)	C24—C25	1.389 (7)
C4—C9	1.405 (7)	C24—H24	0.9300
C4—C5	1.406 (7)	C25—C23^iii^	1.391 (7)
C5—C6	1.353 (7)	C25—H25	0.9300
			
O6—Cd1—O3	120.64 (16)	N1—C11—H11B	109.4
O6—Cd1—O7^i^	102.79 (15)	C12—C11—H11B	109.4
O3—Cd1—O7^i^	89.23 (13)	H11A—C11—H11B	108.0
O6—Cd1—O1	91.71 (16)	C11—C12—H12A	109.5
O3—Cd1—O1	79.38 (13)	C11—C12—H12B	109.5
O7^i^—Cd1—O1	164.82 (13)	H12A—C12—H12B	109.5
O6—Cd1—O5	84.71 (16)	C11—C12—H12C	109.5
O3—Cd1—O5	154.35 (15)	H12A—C12—H12C	109.5
O7^i^—Cd1—O5	88.65 (16)	H12B—C12—H12C	109.5
O1—Cd1—O5	97.27 (15)	C14—C13—N2	111.1 (7)
O6—Cd1—O4	136.90 (15)	C14—C13—H13A	109.4
O3—Cd1—O4	100.57 (14)	N2—C13—H13A	109.4
O7^i^—Cd1—O4	88.88 (14)	C14—C13—H13B	109.4
O1—Cd1—O4	83.45 (15)	N2—C13—H13B	109.4
O5—Cd1—O4	53.83 (14)	H13A—C13—H13B	108.0
C10—N1—C9	119.0 (4)	C13—C14—N3	109.9 (7)
C10—N1—C11	117.2 (4)	C13—C14—H14A	109.7
C9—N1—C11	123.0 (4)	N3—C14—H14A	109.7
C7—N2—C16	122.9 (5)	C13—C14—H14B	109.7
C7—N2—C13	121.7 (5)	N3—C14—H14B	109.7
C16—N2—C13	113.6 (5)	H14A—C14—H14B	108.2
C15—N3—C14	111.3 (5)	C16—C15—N3	110.6 (6)
C15—N3—H3A	124.3	C16—C15—C17	110.9 (8)
C14—N3—H3A	124.3	N3—C15—C17	108.5 (6)
C1—O1—Cd1	132.5 (3)	C16—C15—H15	108.9
C3—O3—Cd1	132.3 (3)	N3—C15—H15	108.9
C18—O4—Cd1	87.0 (3)	C17—C15—H15	108.9
C18—O5—Cd1	95.5 (3)	C15—C16—N2	113.7 (6)
C22—O6—Cd1	114.2 (4)	C15—C16—H16A	108.8
C22—O7—Cd1^i^	129.7 (3)	N2—C16—H16A	108.8
H8A—O8—H8B	108.7	C15—C16—H16B	108.8
H9A—O9—H9B	108.4	N2—C16—H16B	108.8
H5O—O10—H6O	107.2	H16A—C16—H16B	107.7
O1—C1—O2	123.6 (5)	C15—C17—H17A	109.5
O1—C1—C2	119.5 (5)	C15—C17—H17B	109.5
O2—C1—C2	117.0 (5)	H17A—C17—H17B	109.5
C10—C2—C3	118.5 (4)	C15—C17—H17C	109.5
C10—C2—C1	116.5 (4)	H17A—C17—H17C	109.5
C3—C2—C1	125.0 (5)	H17B—C17—H17C	109.5
O3—C3—C2	126.5 (4)	O4—C18—O5	123.0 (5)
O3—C3—C4	117.8 (4)	O4—C18—C19	118.8 (5)
C2—C3—C4	115.6 (4)	O5—C18—C19	118.2 (5)
C9—C4—C5	119.6 (4)	O4—C18—Cd1	66.0 (3)
C9—C4—C3	122.0 (5)	O5—C18—Cd1	57.4 (3)
C5—C4—C3	118.3 (4)	C19—C18—Cd1	170.3 (4)
C6—C5—C4	120.1 (5)	C20—C19—C21^ii^	120.3 (5)
C6—C5—H5	119.9	C20—C19—C18	120.2 (5)
C4—C5—H5	119.9	C21^ii^—C19—C18	119.5 (5)
C5—C6—F1	119.3 (5)	C19—C20—C21	120.1 (5)
C5—C6—C7	123.1 (5)	C19—C20—H20	120.0
F1—C6—C7	117.6 (5)	C21—C20—H20	120.0
N2—C7—C8	121.2 (5)	C19^ii^—C21—C20	119.6 (5)
N2—C7—C6	123.6 (5)	C19^ii^—C21—H21	120.2
C8—C7—C6	115.2 (5)	C20—C21—H21	120.2
F2—C8—C7	116.0 (4)	O6—C22—O7	122.8 (5)
F2—C8—C9	119.7 (5)	O6—C22—C23	117.1 (5)
C7—C8—C9	124.2 (5)	O7—C22—C23	120.1 (5)
N1—C9—C8	124.1 (5)	C25^iii^—C23—C24	119.0 (5)
N1—C9—C4	118.6 (4)	C25^iii^—C23—C22	120.5 (5)
C8—C9—C4	117.3 (5)	C24—C23—C22	120.5 (5)
N1—C10—C2	125.9 (5)	C25—C24—C23	120.5 (5)
N1—C10—H10	117.0	C25—C24—H24	119.8
C2—C10—H10	117.0	C23—C24—H24	119.8
N1—C11—C12	111.1 (5)	C24—C25—C23^iii^	120.5 (5)
N1—C11—H11A	109.4	C24—C25—H25	119.7
C12—C11—H11A	109.4	C23^iii^—C25—H25	119.7
			
O6—Cd1—O1—C1	140.3 (5)	F2—C8—C9—N1	8.1 (8)
O3—Cd1—O1—C1	19.4 (5)	C7—C8—C9—N1	−174.4 (5)
O7^i^—Cd1—O1—C1	−22.5 (9)	F2—C8—C9—C4	−171.3 (5)
O5—Cd1—O1—C1	−134.8 (5)	C7—C8—C9—C4	6.2 (8)
O4—Cd1—O1—C1	−82.7 (5)	C5—C4—C9—N1	177.0 (5)
C18—Cd1—O1—C1	−108.1 (5)	C3—C4—C9—N1	−2.1 (7)
O6—Cd1—O3—C3	−86.7 (5)	C5—C4—C9—C8	−3.5 (8)
O7^i^—Cd1—O3—C3	168.9 (5)	C3—C4—C9—C8	177.3 (5)
O1—Cd1—O3—C3	−1.0 (5)	C9—N1—C10—C2	4.2 (8)
O5—Cd1—O3—C3	83.6 (6)	C11—N1—C10—C2	174.6 (5)
O4—Cd1—O3—C3	80.2 (5)	C3—C2—C10—N1	−0.4 (8)
C18—Cd1—O3—C3	83.7 (5)	C1—C2—C10—N1	−179.1 (5)
O6—Cd1—O4—C18	−22.9 (4)	C10—N1—C11—C12	−99.4 (6)
O3—Cd1—O4—C18	173.7 (3)	C9—N1—C11—C12	70.5 (7)
O7^i^—Cd1—O4—C18	84.7 (3)	C7—N2—C13—C14	−140.9 (7)
O1—Cd1—O4—C18	−108.4 (3)	C16—N2—C13—C14	54.0 (10)
O5—Cd1—O4—C18	−4.4 (3)	N2—C13—C14—N3	−55.0 (9)
O6—Cd1—O5—C18	171.8 (4)	C15—N3—C14—C13	56.4 (9)
O3—Cd1—O5—C18	0.2 (6)	C14—N3—C15—C16	−54.0 (9)
O7^i^—Cd1—O5—C18	−85.2 (4)	C14—N3—C15—C17	−175.8 (7)
O1—Cd1—O5—C18	80.8 (4)	N3—C15—C16—N2	52.3 (9)
O4—Cd1—O5—C18	4.4 (3)	C17—C15—C16—N2	172.7 (6)
O3—Cd1—O6—C22	−54.1 (4)	C7—N2—C16—C15	142.6 (7)
O7^i^—Cd1—O6—C22	42.7 (4)	C13—N2—C16—C15	−52.6 (10)
O1—Cd1—O6—C22	−132.8 (4)	Cd1—O4—C18—O5	7.9 (6)
O5—Cd1—O6—C22	130.1 (4)	Cd1—O4—C18—C19	−170.6 (4)
O4—Cd1—O6—C22	145.0 (3)	Cd1—O5—C18—O4	−8.6 (6)
C18—Cd1—O6—C22	134.0 (4)	Cd1—O5—C18—C19	170.0 (4)
Cd1—O1—C1—O2	149.8 (5)	O6—Cd1—C18—O4	163.4 (3)
Cd1—O1—C1—C2	−30.0 (8)	O3—Cd1—C18—O4	−7.8 (4)
O1—C1—C2—C10	−161.2 (5)	O7^i^—Cd1—C18—O4	−94.2 (3)
O2—C1—C2—C10	18.9 (7)	O1—Cd1—C18—O4	70.6 (3)
O1—C1—C2—C3	20.2 (8)	O5—Cd1—C18—O4	172.1 (6)
O2—C1—C2—C3	−159.6 (5)	O6—Cd1—C18—O5	−8.7 (4)
Cd1—O3—C3—C2	−4.1 (8)	O3—Cd1—C18—O5	−179.9 (3)
Cd1—O3—C3—C4	178.5 (3)	O7^i^—Cd1—C18—O5	93.7 (4)
C10—C2—C3—O3	178.2 (5)	O1—Cd1—C18—O5	−101.5 (4)
C1—C2—C3—O3	−3.2 (9)	O4—Cd1—C18—O5	−172.1 (6)
C10—C2—C3—C4	−4.3 (7)	O6—Cd1—C18—C19	−74 (2)
C1—C2—C3—C4	174.2 (5)	O3—Cd1—C18—C19	114 (2)
O3—C3—C4—C9	−176.7 (5)	O7^i^—Cd1—C18—C19	28 (2)
C2—C3—C4—C9	5.6 (7)	O1—Cd1—C18—C19	−167 (2)
O3—C3—C4—C5	4.1 (7)	O5—Cd1—C18—C19	−66 (2)
C2—C3—C4—C5	−173.5 (5)	O4—Cd1—C18—C19	122 (2)
C9—C4—C5—C6	−1.7 (8)	O4—C18—C19—C20	−166.3 (5)
C3—C4—C5—C6	177.5 (5)	O5—C18—C19—C20	15.0 (8)
C4—C5—C6—F1	−174.0 (5)	Cd1—C18—C19—C20	76 (2)
C4—C5—C6—C7	4.7 (9)	O4—C18—C19—C21^ii^	17.0 (8)
C16—N2—C7—C8	−50.8 (9)	O5—C18—C19—C21^ii^	−161.7 (5)
C13—N2—C7—C8	145.6 (7)	Cd1—C18—C19—C21^ii^	−101 (2)
C16—N2—C7—C6	130.3 (7)	C21^ii^—C19—C20—C21	−0.5 (9)
C13—N2—C7—C6	−33.4 (10)	C18—C19—C20—C21	−177.1 (5)
C5—C6—C7—N2	176.7 (6)	C19—C20—C21—C19^ii^	0.5 (9)
F1—C6—C7—N2	−4.5 (9)	Cd1—O6—C22—O7	8.1 (7)
C5—C6—C7—C8	−2.3 (9)	Cd1—O6—C22—C23	−173.2 (3)
F1—C6—C7—C8	176.5 (5)	Cd1^i^—O7—C22—O6	−109.6 (5)
N2—C7—C8—F2	−4.8 (8)	Cd1^i^—O7—C22—C23	71.8 (6)
C6—C7—C8—F2	174.2 (5)	O6—C22—C23—C25^iii^	−155.0 (5)
N2—C7—C8—C9	177.6 (5)	O7—C22—C23—C25^iii^	23.7 (7)
C6—C7—C8—C9	−3.4 (8)	O6—C22—C23—C24	24.4 (7)
C10—N1—C9—C8	177.8 (5)	O7—C22—C23—C24	−156.9 (5)
C11—N1—C9—C8	8.1 (8)	C25^iii^—C23—C24—C25	1.0 (9)
C10—N1—C9—C4	−2.8 (7)	C22—C23—C24—C25	−178.4 (5)
C11—N1—C9—C4	−172.5 (5)	C23—C24—C25—C23^iii^	−1.0 (9)

Symmetry codes: (i) −*x*+1, −*y*+1, −*z*+1; (ii) −*x*+2, −*y*+2, −*z*+2; (iii) −*x*, −*y*+1, −*z*+1.

### Hydrogen-bond geometry (Å, °)

**Table:**

*D*—H···*A*	*D*—H	H···*A*	*D*···*A*	*D*—H···*A*
N3—H3A···O8	0.86	2.33	2.749 (8)	110
O8—H8A···O2^iv^	0.85	2.02	2.854 (7)	166
O8—H8B···O4^v^	0.85	2.01	2.837 (7)	166
O9—H9B···O10^vi^	0.85	1.84	2.687 (14)	178
O9—H9A···O5^vii^	0.85	1.93	2.784 (9)	178
O10—H5O···O2	0.85	2.13	2.971 (10)	171
O10—H6O···O9	0.85	2.42	2.957 (14)	121

Symmetry codes: (iv) −*x*+2, −*y*, −*z*+1; (v) *x*, *y*−1, *z*−1; (vi) −*x*+1, −*y*, −*z*+2; (vii) *x*, *y*−1, *z*.
